# Investigation of rice performance characteristics: A comparative study of LR, ANN, and RSM

**DOI:** 10.1002/fsn3.2953

**Published:** 2022-07-02

**Authors:** Mandana Mahfeli, Mohammad Zarein, Aliasghar Zomorodian, Hamid Khafajeh

**Affiliations:** ^1^ Biosystems Engineering Department Tarbiat Modares University Tehran Iran; ^2^ Department of Biosystems Engineering Shiraz University Shiraz Iran

**Keywords:** breakage resistance, central composite design, head rice yield, optimization, radial basis function

## Abstract

Parboiling is a type of heat pretreatment used in rice processing to reach higher head rice yield and improve the nutrition properties of raw rice. In this research, the goals were prediction and determination of optimum conditions for parboiled rice processing using the response surface method (RSM) as well as modeling the output values by linear regression (LR) and artificial neural networks (ANN). The parameters including steaming time (0, 5, 10, and 15 min), dryer type (solar and continuous dryers), and drying air temperature (35, 40, and 45°C) were employed as input values. In addition, the breakage resistance (BR) and head rice yield (HRY) were selected as output values. The ANN‐based nonlinear regression, the multi‐layer perceptron (MLP), and the radial basis function (RBF) have been developed to model the process parameters, as well as the central composite design (CCD) was conducted for optimization of BR and HRY values. The outputs of RBF network have been successfully applied to predict higher coefficient of determination of BR and HRY as 0.989 and 0.986, respectively, indicating the appropriateness of the model equation in predicting head rice yield and breakage resistance when the three processing variables (steaming time, dryer type, and drying air temperature) are mathematically combined. Also, the lower root mean square error (RMSE) was obtained for each one as 0.043 and 0.041. The optimum values of BR and HRY were obtained as 12.80 N and 67.3%, respectively, at 9.62 min and 36.9°C for a solar dryer with a desirability of 0.941. In addition, the same values were obtained as 14.50 N and 72.1%, respectively, at 8.77 min and 37.0°C for a continuous dryer with a desirability of 0.971.

## Graphical abstract

Parboiling is a type of heat pretreatment used in rice processing to reach higher head rice yield and improve nutrition properties of raw rice. In this research, the goal was prediction and determination of optimum conditions for parboiled rice processing using response surface method (RSM) as well as modeling the output values by linear regression (LR) and articial neural networks (ANN).

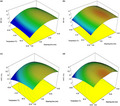

## 1 INTRODUCTION

Rice is a precious and popular food crop in the diets of about 75% of the world's populations (Soponronarit et al., 2004). Rice grains contain various minerals, proteins, and vitamins, which may be lost in the processing steps. Parboiling is an effective way as a pretreatment in rice drying process that preserves the nutritional properties of grain to an acceptable extent (Jannasch & Wang, 2020). The physical properties of the milled rice grains play a critical role in determining their market value. Properties like head rice yield, breakage resistance of kernels, broken rice ratio, grain hardness, and color are major determinants of milled rice acceptability by consumers (Campbell et al., 2009). During the processing and milling operation, rice kernels seem to be prone to crack which is uncontrollable to prevent in order to obtain the optimum head rice yield (HRY). Parboiling pretreatment causes a higher head rice yield with assumed minimal damage to grains by gelatinization of rice starch during processing time. This may be due to the role of gelatinization of starch in filling the voids and fissures (Hapsari et al., 2016). By measuring the three‐point bending strength in some researches, it was found a significant relationship between the physical properties and HRY of rice and also a strong relationship between HRY and the percentage of kernels that could tolerate certain breakage force (Qi et al., 2003). There have been many studies on optimizing the processing conditions to limit waste and improve the HRY during the conversion of paddy (Aquerreta et al., 2007; Jindal & Siebenmorgen, 1987; Mukhopadhyay et al., 2019; Siebenmorgen et al., 2004; Steffe & Singh, 1980). Artificial neural networks (ANNs) are an attractive mathematical tool that potentially is configured for engineering purposes, such as pattern recognition, forecasting, and data compression. Multi‐layer perceptron (MLP) network and radial basis function network (RBF) are the most common architectures of ANN (Raj & David, 2020). MLP network as a supplement of feed‐forward ANN is composed of input, hidden, and output layers. The RBF in its simplest form is a three‐layer feed‐forward neural network that uses radial basis functions as activation functions of the inputs and neuron parameters (Venkateswarlu & Jujjavarapu, 2019). Presently, a well‐trained ANN offers exciting possibilities to modeling and prediction in different fields of agriculture such as prediction of crop yield, seeding dates, and biomass production. The ANN is abundantly utilized to simulate various processes, particularly for some cases where other statistical modeling fails. Recently, there has been an increasing desire to apply ANN in agriculture due to faster prediction and possibility of adding or removing input and output variables compared to other conventional statistical models (Ghamari et al., 2010). Some researchers applied ANN models to estimate physical and physiological damage to seeds, determine the sugar content in fruits, estimate the crop yield, and moisture ratio of kernels during soaking. Results indicated that ANN models are the best methods for prediction because of its ability to modeling and classification in biological fields with more acceptable accuracy compared to other models (Kashaninejad et al., 2009; Khairunniza‐Bejo et al., 2014; Khazaei et al., 2008; Liu et al., 2001; Oda et al., 2012; Saad & Ismail, 2009). In many works, researchers compared regression models and ANN to predict the crop yield and quality (Kim, 2008; Kumar, 2020; Stangierski et al., 2019). The response surface methodology (RSM) is a powerful mathematical modeling tool with a collection of empirical statistical techniques that is widely employed to find optimum conditions in varied processes to solve multivariable equations simultaneously by performing a minimal number of experimental runs (Betiku & Adesina, 2013; Danbaba et al., 2014; Karuppaiya et al., 2010; Mason et al., 2003). Danbaba et al. (2014) employed RSM involving central composite design (CCD) to study the effects of soaking temperature, steaming time, and drying time on the HRY of parboiled rice. They concluded that the research was conducted at the National Cereals Research Institute, Badeggi, Nigeria. Results indicated that regression coefficient of the developed model was significant (*F*‐value 16.33 and *p*‐value .003) indicating that most of the variation in head rice yield can be explained by the regression model. Coefficient of regression *R* ^2^ and adjusted *R* ^2^ were .97 and .91, respectively, indicating appropriateness of the RSM and CCD model in predicting optimum rice parboiling condition for maximum head rice recovery (Danbaba et al., 2014). In a similar study, RSM was successfully applied to optimize the processing conditions in grain production (Ghodke et al., 2009; Ogunbiyi et al., 2018). Despite numerous studies about the use of RSM and ANN strategy in several food research studies, scarce information has been reported on the application and comparison of mathematical models to predict HRY and BR of grains (in particular parboiled rice) under different processing conditions. The traditional method of studying one modeling method at a time can be effective in some cases, but it will be more useful to consider combined methods of possible model predicting and optimizing effective parameters for biological or physical processes. The RSM, LR, and ANN methods, which are based on statistical principles, can be applied as tools to implement process improvement strategy that will drive optimal HRY and BR from a given paddy lot by performing a minimal number of experiments. In addition, optimizing of process using RSM in combination with factorial experimental design of Box–Behnken design is essential for fitting a quadratic surface, which works well for process optimization. It has been investigated the impact of various parboiling processing conditions on rice characteristics by many researchers (Alkhafaji et al., 2020; Hunt, 2019; Likitrattanaporn & Noomhorm, 2011; Messia et al., 2012). Nevertheless, modeling and optimization of the processing of parboiled rice can improve the yield and its characteristics qualities. Meanwhile, different tools can be employed in modeling and optimization of experimental data for parboiling process. Such tools include RSM and ANN design, to mention but a few. In previous studies, researchers have reported the use of one or two software applications (Dash & Das, 2019; Dash & Das, 2021). However, the use of the combination of all three RSM, ANN, and LR methods, in modeling and optimization of parboiling process of paddy (in particular Hashemi paddy cultivar) has received little or no attention from the researchers. Its main advantage is the ability to compare statistically various results obtained from each of the above‐mentioned software applications and eliminate the disadvantages of using a single one. In addition, the comparison among three methodologies shows certain differences in overall accuracy, sensitivity, and optimal result.

Various parameters and evaluating “one‐variable‐at‐a‐time” could be time consuming, expensive, and inefficient. Thus, application of process modeling approaches including RSM and ANN is required and beneficial for optimization and modeling of paddy parboiling conditions. As rice kernels are very fragile during processing, determining the parboiling condition is very critical and needs great optimization and care. The main goal of the present study was to understand and compare the topography of the different methods (RSM, LR, and ANNs) in terms of fitting quality and optimization, to find a region where the most appropriate response occurs. A similar comparing study that was specifically developed to determine the maximum HRY and BR for Hashemi paddy cultivar parboiled in different conditions of steaming time (zero, 5, 10, and 15 min), dryer type (solar and continuous dryers), and inlet drying air temperature (35, 40, and 45°C) has not yet been reported.

## 2 MATERIALS AND METHODS

**FIGURE 1:**
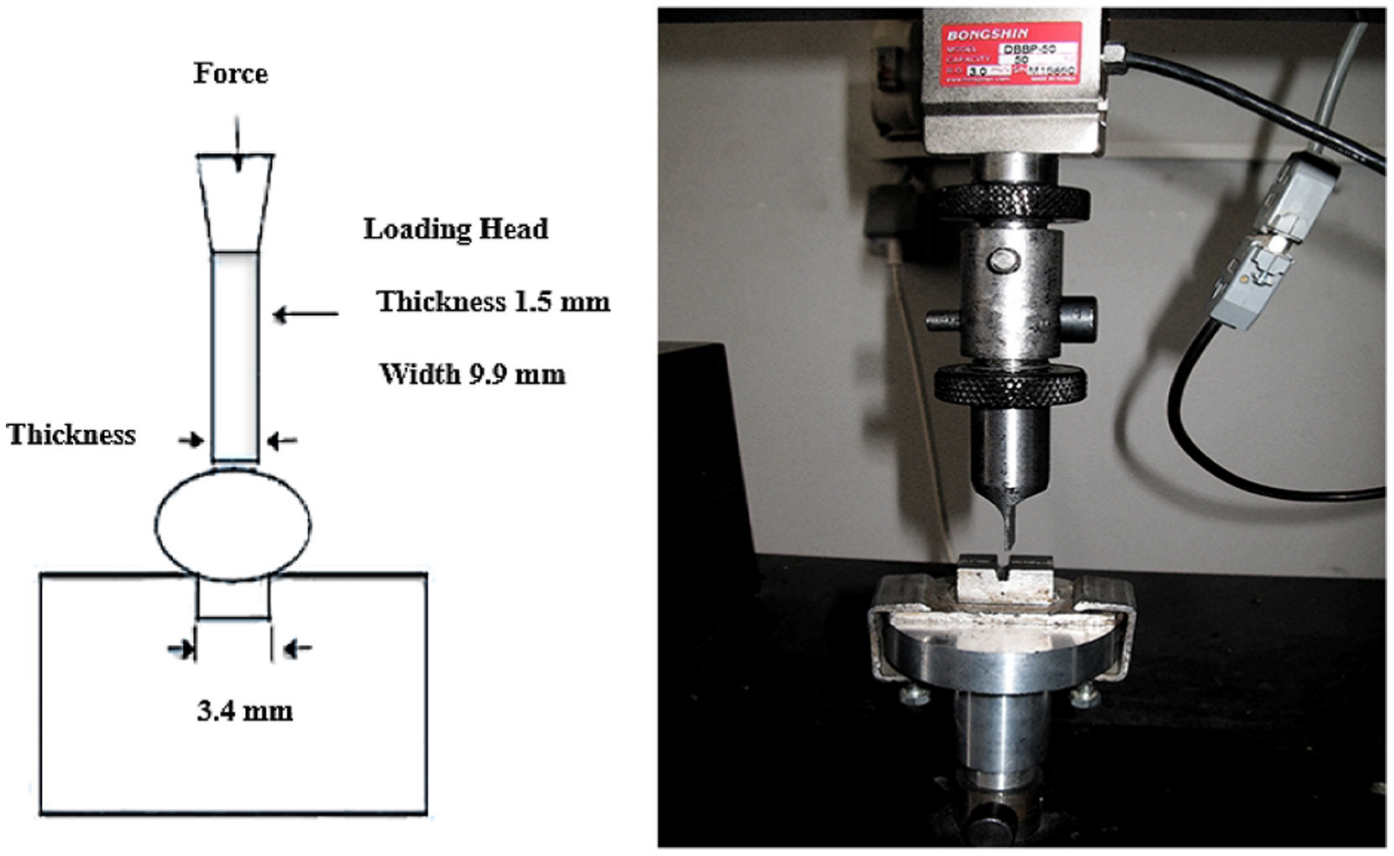
Measuring the breakage resistance of rice in three‐point bending test using Instron machine

The Iranian local rice cultivar of Hashemi, which is classified as a tall grain rice, was applied for parboiling experiments at Biosystems laboratory, University of Shiraz. After the harvesting process, the paddy was found to contain an initial moisture content of approximately 28 ± 1 (% w.b.). Before the different steps of experiments were done, sealed plastic bags were used to keep the rice samples that have been stored at 4 ± 1°C in a refrigerator. In this study, parboiling of paddy grains was accomplished by the conventional method that consists of soaking, steaming, and drying processes. Paddy samples were soaked in hot water at 80°C for 1 h. Open steaming step was then conducted following draining of water. Two drying modes of passive solar and continuous method were employed for drying. Following the drying step, paddy grains were subjected to open aeration at room temperature for 1 week to reach a final moisture content of 11.5 ± 1°C. A testing rubber roll huller (Satake THU‐35A, Japan) was then applied to dehusk the dried paddy samples. Many studies on the mechanical properties of grains reported that bending strength and fracture energy are a proper criterion to determine the performance of rice kernels (Lu & Siebenmorgen, 1995). On the other hand, due to difficulty of tensile strength tests for rice, the best option for testing is bending test (Nassiri & Etesami, 2015; Zhang et al., 2005). A three‐point bending test was conducted by the Instron Testing Machine (STM‐20 SANTAM, Iran) with a loading rate of 10 mm/s. In order to evaluate the sample breakage resistance in bending, two types of raw and parboiled dehusked samples (per type of 100 g of grain) were isolated by random selecting and then loaded by Instron jaw blades (Figure 1).

Generally, the HRY was introduced as weight percentage of grains three‐fourths or more of whole grain (in terms of head rice yield) or weight percentage of husked paddy (in terms of rice‐processing yield) to the total weight of milled rough rice (Farounk & Islam, 1995). In order to significantly improve the accuracy and acceleration of model performance, data value normalization was accomplished as follows (Lallahem & Mania, 2003):

(1) $$X_{\mathrm{norm}}=\frac{x-x_{\min}}{x_{\max}-x_{\min}}$$

where *X*_norm_ = normalized value, *x* = observed value, *x*_min_ = minimum values, and *x*_max_ = maximum values.

The measured data from these experiments were then used to optimize the design with all three RSM, ANN, and LR models with the objective of a maximal HRY and BR. Statistical comparison between the targets and predicted parameters was made using root mean square error (RMSE), mean absolute percentage error (MAPE), and the coefficient of determination (*R* ^2^).

### 2.1 Linear regression (LR) model

Regression analysis is an effective statistical technique for examining parboiling pretreatment effects on mechanical properties of rice kernels. This approach is applied as a tool to estimate and model linear relationships (denoted by a best‐fitted straight line) between variables and predict optimum response values using mathematical equations. Conventionally, the LR model is expressed as:

(2) $$y=b_{0}+b_{1}x_{1}+\ldots +b_{n}x_{n}+e$$

where *y* = value of the dependent variable, *x*_*n*_ = predictor variable, *b*_*n*_ = coefficient value, *e* = observed error (uncontrolled factors and experimental error). The (*b*_*j*_) is model parameters determined by a regression model.

### 2.2 Artificial neural network (MLP, RBF) models

**FIGURE 2:**
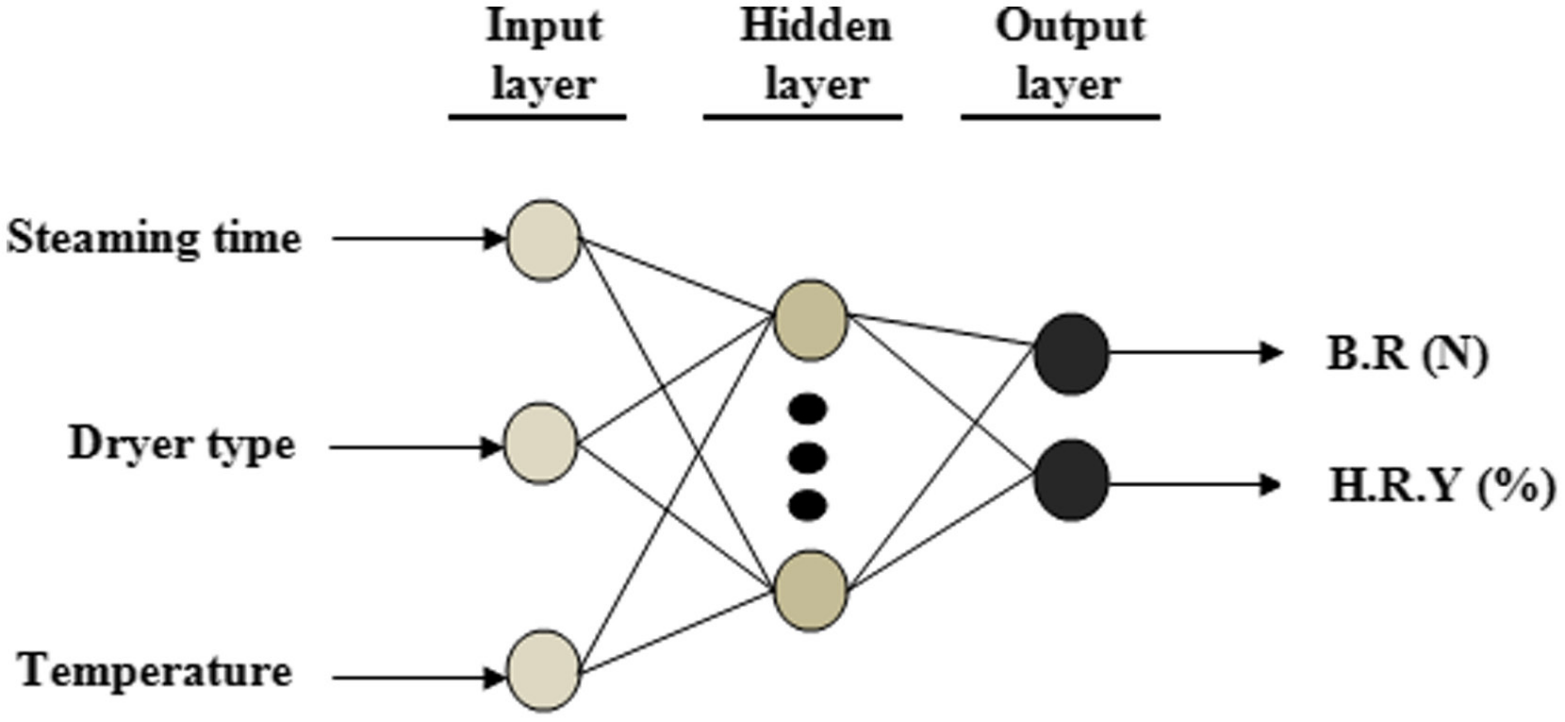
MLP and RBF neural network structure used in the study

Neural networks can be applied as a direct substitute for autocorrelation, multivariable regression, LR, trigonometric, and other statistical analysis and techniques (Singh et al., 2003). Neural networks with respect to its unique aspects for pattern identify from complicated data can be used to present solutions in complex problems that may not be previously applied by common computer methods. A trained neural network can be professionally exposed and modeled a set of categorized data to compare, simulate, optimize, and analyze response variables in terms of favorite situations. In addition, a multi‐layer network technique detects best patterns using information sets for data mining and forecasting. In order to expose optimal outputs of network, the main premise is selection of Neuron Model (Single‐Input Neuron, Transfer Functions, and Multiple‐Input Neuron) and Network Architectures (A Layer of neurons, Multiple Layers of Neurons, and Recurrent Networks) (Simpson, 1991). Multi‐layer perceptron and radial basis function neural networks, as two of the neural architecture of ANN networks, can be used to simulate process outputs of regression problems with high accuracy (Kumar & Yadav, 2011; Lim et al., 2000). Radial basis function (RBF) compared with multi‐layer perceptron (MLP) is responsive only to a limited part of input space, but MLP has more distributed approach. In present study, the predictive performance of two different ANN architectures (MLP and RBF) was applied to the estimation of the BR and HRY. For the purpose of this study, the toolbox of ANN was applied to predict the nonlinear relationship between the input variables (steaming time, dryer type and drying air temperature) and the outputs (HRY and BR). The schematic of the ANN model used is presented in Figure 2. The data employed for experimental study were randomly divided into three groups: 70% in the training set, 15% in the validation set, and 15% in the test set. The structure of the three‐layer feed‐forward network studied in this paper was built using three input variables and two output variables to select the best predictive model. Feed‐forward back‐propagation and algorithm of Levenberg–Marquardt (TRAINLM) were used in developing the ANN architecture. Different number of neurons in the hidden layer ranging from 1 to 16 neurons and transfer function (Tansig and Logsig) was applied based on the trial method to develop the optimum ANN model that can minimize the deviations between the predicted and experimental results. Also, the output signal for accuracy was determined by modifying the number of layers, weight sum of input variables, and bias during algorithm iterative until the ANN outputs were closer to the actual values. The best training performance of the developed ANN model was terminated at the lowest RMSE and highest determination coefficient (*R* ^2^) for training set. Sanusi and Akinoso (2021) also reported a similar approach while modeling impact of process variables on brown rice quality and overall energy consumption (Sanusi & Akinoso, 2021).

### 2.3 Multi‐layer perceptron (MLP) model

The MLP neuron network is known as a common architecture for neural networks. The MLP consists of node (neuron) layers (an input layer, a hidden layer, and an output layer). The MLP model used activation function in all neurons to obtain an output by mapping of weighted sum of the inputs and bias terms. This arrangement presents a structure with feed‐forward layered topology known as feedforward ANN (Alexandridis & Chondrodima, 2014). The MLP network is back propagated from an output layer to one or more hidden layers and, eventually, to an input layer, with a number of neurons in each layer. It was known the neuron connection between the input layers and hidden layers as the input weight matrix and neuron relationship between hidden layers and output layers is identified as the output weight matrix (Zhao et al., 2009). Each node or neuron, after calculating the sum of weighted input signals (*x*_*j*_, for *j* = 1, 2,…, *n*), creates a nonlinear activation function to generate output signal y as (Zarein et al., 2019):

(3) $$y=\varphi \left(\sum_{j=1}^{n}w_{i}x_{j}\right)$$

The function of Sigmoidal feedforward artificial neural networks that satisfies this criterion is expressed as (Dawson & Wilby, 1998):

(4) $$y=\frac{1}{1+\exp\left(-\sum_{j=1}^{n}w_{i}x_{j}\right)}$$

By comparison of neural network error measures, we can estimate the performance of the network. Error can be determined based on the difference between the targeted and predicted outputs. The error function can be evaluated as in the study cited herein (Zarein et al., 2019):

(5) $$E=\frac{1}{P}\sum_{P}\sum_{K}{\left(z_{\mathrm{pk}}-{\overset{´}{z}}_{\mathrm{pk}}\right)}^{2}$$

where *k* = element index in the output vector, *z*_*pk*_ = the *k* ^*th*^ element of the *p* ^*th*^ target pattern vector, *p* = the training pairs index of vectors, and *z'*_*pk*_ = the *k* ^*th*^ element of the predicted vector when pattern *p* is expressed as input to the network.

### 2.4 Radial basis function (RBF) neural network

In the field of linear and nonlinear data modeling, RBF model is based on supervised learning that commonly uses radial basis functions as activation functions. RBF networks is a popular alternative because of its mathematical simplicity, the computations relatively cheap, and also quick learning (learning in one stage) of the given application (Alexandridis & Chondrodima, 2014). This RBF neural network structure is similar to the MLP model except that it has a hidden layer with nodes as RBF units. Two main parameters of RBF model are location of the function's center and its deviation or width. The hidden unit determines the distance between an input data vector and the center of its RBF. The RBF gradually increased to a peak when the distance between the input vector x and its center vector declines to zero value. The output of the weights connecting the hidden layer to the output layer is a linear combination of RBFs of the inputs and neuron parameters that its processing is rapid (Foody, 2004). The output of RBF network is:

(6) $$y_{k}=\sum_{j=1}^{M}w_{\mathrm{kj}}\emptyset_{j}\left(x\right)+w_{k0}$$

where *M* = the number of basic functions, *x* = the input data vector, *w*_*kj*_ = a weighted connection between the basis function and output layer, and *Ø*_*j*_ = the nonlinear function of unit *j*, which is typically a Gaussian form.

(7) $$\emptyset_{j}\left(x\right)=\exp\left(-\frac{{\left|x-\right.\left.\mu_{j}\right|}^{2}}{2{\sigma_{j}}^{2}}\right)$$

where *x* = input of RBF unit, *μ* = the center of RBF unit, and *σ*_*j*_ = the spread of the Gaussian basis function. Optimization of weights is done by least mean square (LMS) algorithm once the centers of RBF units are determined. In this study, centers were randomly selected from the data set.

### 2.5 Response surface methodology (RSM)

The RSM is an efficient procedure widely used for designing, optimizing, developing, and analyzing new scientific and existing products. The RSM presents criteria to evaluate the effect of independent variables, alone or in combination, on processes. In addition, the RSM makes it possible to predict the most precise response using compilation of mathematical methods (Farooq Anjum et al., 1997; Halim et al., 2009). In order to determine the optimum value, Equation (8) is applied:

(8) $$Y_{i}=\beta_{0}+\sum \beta_{i}X_{i}+\sum \beta_{\mathrm{ij}}X_{i}X_{j}+\sum \beta_{\mathrm{jj}}{X_{i}}^{2}+\varepsilon$$

where *ß*_*0*_ = regression coefficients for intercept, *ß*_*i*_ = linear coefficients, *ß*_*ij*_ = interaction coefficients.

*ß*_*jj*_ = quadratic coefficients, *X*_*i*_ and *X*_j_ = coded independent variables and ε = error.

In this study, central composite design (CCD) was carried out with 22 run and three center points. The performances of the LR, MLP, RBF, and RSM models were compared using statistical parameters of *R* ^2^, MAPE, and RMSE as follows (Zarein et al., 2015):

(9) $$R^{2}=1-\left(\frac{\sum_{i=1}^{n}{\left(z-\overset{´}{z}\right)}^{2}}{\sum_{i=1}^{n}{z_{i}}^{2}}\right)$$

(10) $$\text{MAPE}=\frac{1}{N}\sum_{i=1}^{N}\left|\frac{z-\overset{´}{z}}{z}\right|\times 100$$

(11) $$\text{RMSE}=\sqrt{\frac{\sum_{i=1}^{n}{\left(z-\overset{´}{z}\right)}^{2}}{N}}$$

where *z* = the measured value, *z'* = the predicted value, *N* = the total number of observations (Armaghani et al., 2015; Garg et al., 2015).

*R*‐squared (*R* ^2^) value is a statistical measure that is commonly between zero and one, and illustrates the ability of a parameter to predict another parameter. The maximum value for MAPE (100) and the minimum value for RMSE (0) define the highest values for model performance. The MAPE is an index of accuracy ratio versus size of project that can be indicated based on percentage that can measure forecast accuracy. Good performance of the model is based on minimizing MAPE (Khoshnevisan et al., 2014). The present study used RSM combined with central composite design to investigate the effects of three identified factors (steaming time, dryer type, and inlet air drying temperature) on the response of paddy to parboiling process. Data were analyzed using IBM SPSS Statistics version 18.0 (IBM), STATISTICA version 12.0 (StatSoft Inc.), and Design‐Expert version 7.0.0.1 (Stat‐Ease Inc.).

## 3 RESULTS AND DISCUSSION

### 3.1 Linear regression model

HRY and BR are among the key quality indices used by rice processors. Parboiled rice with high values of these factors could signify rice with potential market value. In this study, BR and HRY were considered as a function of steaming time, dryer type, and drying air temperature. Table 1 tabulates the coefficient of this function with t‐ratio value and regression results for Equations 12 and 13. Results show that in terms of BR, it was detected significant changes between steaming times (*p* < .01), dryer type, and inlet drying air temperature (*p* < .05). With respect to the obtained coefficients, it was known that an additional use of 1% for each of these inputs would lead to 1.092 increase in BR. The impacts of steaming time (*p* < .05), dryer type (*p* < .01), and inlet drying air temperature (*p* < .01) are statistically significant for HRY. The LR models for the prediction of BR (Equation [12]) and HRY (Equation [13]) are:

(12) $$\mathrm{BR}=\left(0.225\right)\;S.T+\left(-0.159\right)\;\text{Dryer type}+\left(-0.182\right)\;\text{Temperature}+0.491$$

(13) $$\mathrm{HRY}=\left(-0.109\right)\;S.T+\left(-0.187\right)\;\text{Dryer type}+\left(-0.454\right)\;\text{Temperature}+0.846$$

**TABLE 1:** Model summaries of the liner regression models for BR and HRY prediction

Independent variables	BR			HRY		
	Coefficient	Std.error	*t*‐value	Coefficient	Std.error	*t*‐value
Constant	0.491	0.072	6.827**	0.846	0.048	17.722**
Steaming time	0.225	0.079	2.864**	−0.109	0.052	−2.084*
Dryer type	−0.159	0.064	−2.479*	−0.187	0.043	−4.386**
drying air temperature	−0.182	0.079	−2.307*	−0.454	0.052	−8.679**

*Note:* **^,^* Significant at 1 and 5% probability level, respectively.

**FIGURE 3:**
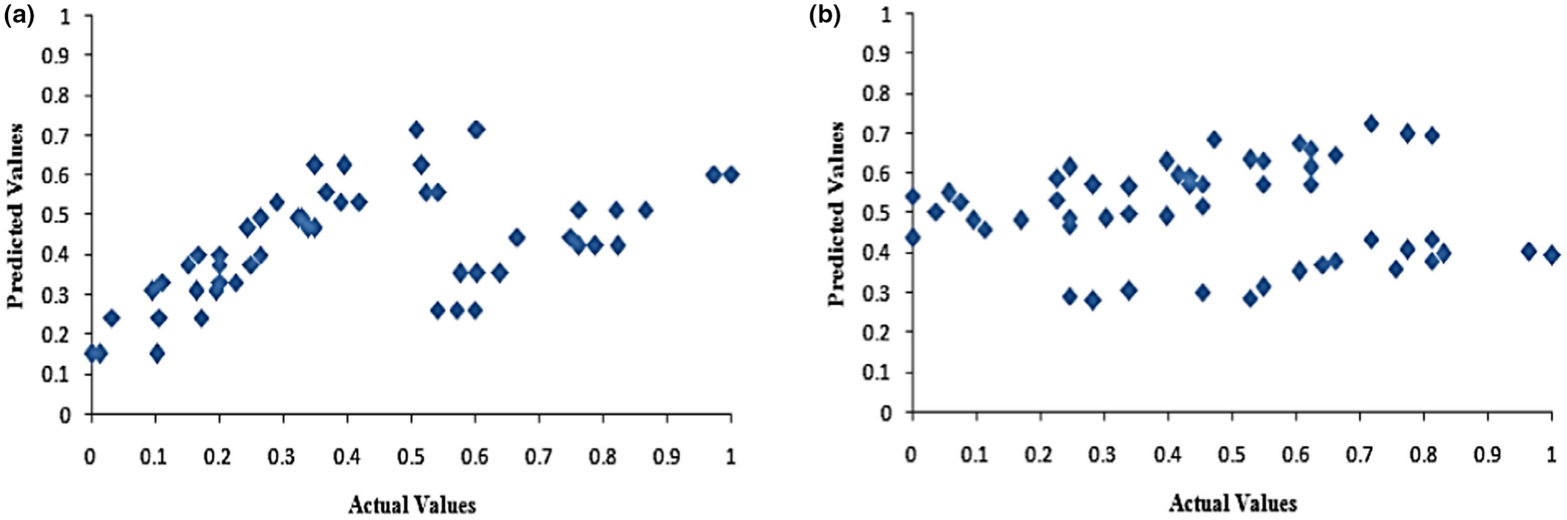
Cross‐correlation of predicted and actual values of BR (a) and HRY (b) for linear regression model

*R*‐squared (*R* ^2^) was applied to estimate the model's predictive performance for the measured and predicted values. Development of LR model for BR (*R* ^2^ = .282) and HRY (*R* ^2^ = .664) illustrated unsuitable correlation for the relationships between the measured and predicted values as shown in Figure 3. A regression model in the form of a linear model was developed by Rao et al. (2007) that was related to all the variables in the thin layer drying experiments for parboiled rice. The linear model fitted well to the head rice yield values with a much higher coefficient of determination (*R* ^2^ = .98). It was found from ANOVA that the temperature, velocity, and bed depth had a significant effect on HRY at the 5% level (Rao et al., 2007).

### 3.2 MLP model

**FIGURE 4:**
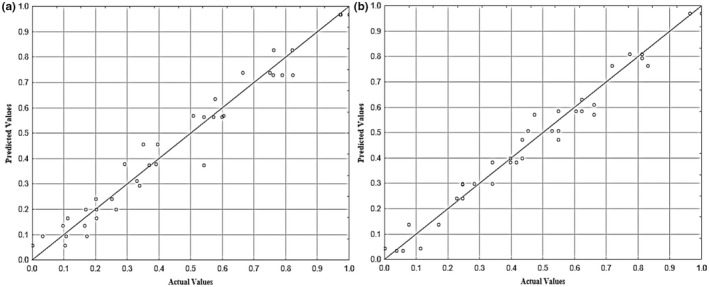
Cross‐correlation between predicted and actual values of BR (a) and HRY (b) for MLP

In this study, several multi‐layer perceptron networks with one or two layers and with 1–20 neurons were executed, trained, validated, and generalized to determine the best function. It was chosen an efficient approach based on MLP Back Propagation Neural Network to build the prediction models. MLP model runs by different steaming time, dryer type, and drying air temperature as input variables and the BR and HRY as output variables. In the case of the final chosen model of MLP, the most suitable structure with one input layer, three input variables, one hidden layer with 7 neurons, and one output layer with one output variable (3‐7‐1 structure) was selected. The predictive capability of the generated ANN models for HRY and BR was tested using unknown set of inputs data, and the predicted values and experimental values were plotted for HRY and BR as shown in Figure 4. With respect to observed cross‐correlation among predicted and target values, it could be detected that MLP model was effective for the prediction HRY and BR of parboiled rice. Table 2 indicates *R* ^2^, RMSE, and MAPE values for prediction of models. Results exhibited satisfactory prediction performance for MLP method. The *R* ^2^ between the ANN experimental and predicted data for HRY and BR was 0.983 and 0.981, respectively. This result showed that the predictive accuracy of the ANN model for both output variables was high.

**TABLE 2:** Performance indices (*R* ^2^, RMSE, and MAPE) for different models

Model	BR			HRY		
	*R* ^2^	RMSE	MAPE (%)	*R* ^2^	RMSE	MAPE (%)
LR	.282	0.227	86.859	.664	0.293	104.382
ANN‐MLP	.981	0.055	18.381	.983	0.045	10.492
ANN‐RBF	.989	0.043	17.583	.986	0.041	10.331

Behroozi‐Khazae et al. previously investigated the possibility of application of the ANN approach with K‐fold cross‐validation along with the MVR to create reliable model of parboiling process of an Iranian rice variety with a small dataset. The highest value of *R* ^2^ and lowest value of MSE for each MQ variables showed that the K‐fold cross‐validation and the ANN model can be used for modeling and predicting quality parameters of parboiling rice (Behroozi‐Khazaei & Nasirahmadi, 2017).

### 3.3 RBF model

**FIGURE 5:**
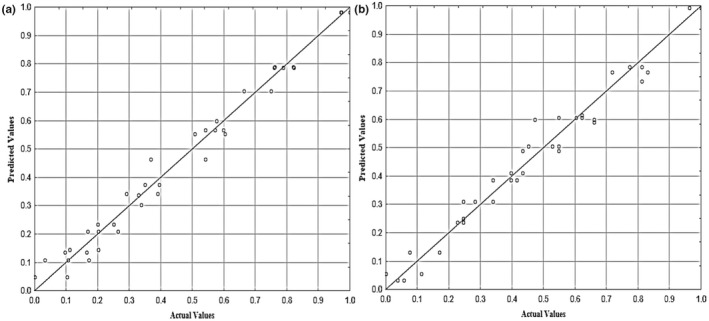
Cross‐correlation between predicted and actual values of BR (a) and HRY (b) for RBF

The RBF used a single‐layer neural network to determine and model the network parameters. The transformation of input to output of the hidden layer cells takes place directly. This output cells multiplied by the weights entered a specific collector as the output of the neural network. Data used for network training were the one applied to generalize the MLP network. Actual values of the predicted BR and HRY variables are presented in the following scatter plots (Figure 5). *R* ^2^, RMSE, and MAPE are indicated in Table 4. Based on achieved parameters of the final chosen model, the RBF model showed higher acceptability for the prediction of rice HRY (*R* ^2^ = .986; RMSE = 0.041) and BR (*R* ^2^ = .989; RMSE = 0.043) than that of the MLP ones. MLP, RBF, and ANFIS networks had been investigated to determine and predict percentage of swell of soil (S%) by Yilmaz and Kaynar. Comparison of the results with the same by a multiple regression (MR) model showed that RBF performed much better than MLP, ANFIS, and MR for predicting the S percentage with lowest MAPE and RMSE and the highest *R* ^2^ (Yilmaz & Kaynar, 2011).

### 3.4 Comparison of ANN models

The main objective of development of LR and ANN (MLP‐RBF) models in this study was performance comparison of models to predict optimal BR and HRY. The observed results from linear regression analysis indicated that relationship between the output and input parameters is statistically unacceptable. The prediction models were developed with three inputs and two outputs and the networks were independently evaluated for each output. The analytical processes of models for prediction of BR and HRY showed that the obtained equations from the MLP model provided relatively satisfactory prediction performance. The predicted values by the RBF model with three inputs and one output exhibited higher reliability compared to the ones predicted by the LR and MLP models. In addition, the *R* ^2^, RMSE, and MAPE values showed that the performance of RBF model was better than LR and MLP models for prediction. Most of the published papers reported that the RBF models outperformed the other models (Wu et al., 2004).

### 3.5 Optimization and validation using RSM

A central composite design was successfully employed in this study to develop a relationship between rice performance characteristics (BR and HRY) and independent variables (steaming time, dryer type, and drying air temperature) in order to maximize the BR and HRY. The BR and HRY for both dryers was varied from 5.34 to 15.90 N and from 29% to 70%, respectively. The maximum BR (15.14 N) and HRY (70%) were obtained at steaming time of 10 min by continuous dryer and at drying air temperature of 40°C. The advanced multiple regression analysis was conducted to determine the polynomial equation with the full regression model coefficient. The following equations present significant terms extracted from the coded form of model:

(14) $$\mathrm{BR}=+12.98+1.01A-0.88B-0.76C-0.00\text{5AB}+0.17\mathrm{BC}-4.82A^{2}$$

(15) $$\mathrm{HRY}=+0.61–0.03\text{2A}-0.55B-0.13C-0.00\text{2AB}-0.00\text{4BC}-0.11A^{2}$$

where BR and HRY are breakage resistance and head rice yield, respectively. *A, B,* and *C* are the coded forms of steaming time (min), dryer type, and drying air temperature (°C), respectively. From the equation, the coefficient with one factor signifies the effect in an individual form while the coefficient which has two factors and second‐order form signifies the interaction between them and their fourth route effect. The suffix symbols positive or negative (+/−) signifies the synergy and antagonistic effects, where the positive stands for synergistic effect and the negative stands for antagonistic effect (Joshi et al., 2008). Then the model was investigated by analysis of variance (ANOVA) that was conducted for fitting the model using Design‐Expert software. ANOVA is an effective statistical method, which bifurcates into individual roots and allows user to find the sum of all the data variation in the model with specific sources of variation (Srikanth et al., 2018). Thus, the model variation is given in Table 3.

**TABLE 3:** Experimental process obtained for rice samples

Run	Steaming time (min)	Dryer type	Drying air temperature (°C)	BR (*N*)		HRY (%)	
				Predicted	Obtained	Predicted	Obtained
1	15	1	40	10.04	7.49	63.48	56.30
2	0	1	40	10.93	10.71	52.69	47.70
3	15	1	45	8.81	6.61	36.10	28.70
4	10	1	35	8.62	8.14	33.67	30.70
5	10	1	40	3.81	5.34	36.58	40.70
6	15	1	35	10.70	9.70	51.38	53.00
7	5	1	45	12.61	15.14	66.32	79.00
8	5	1	35	10.84	13.28	43.12	54.00
9	10	1	40	12.65	12.50	62.92	62.00
10	10	1	45	12.65	12.50	62.92	59.00
11	10	1	40	12.65	12.90	62.92	61.00
12	10	2	40	12.04	12.38	71.49	67.70
13	10	2	40	12.17	13.95	52.49	57.00
14	5	2	45	11.57	11.69	42.09	40.30
15	10	2	40	10.61	12.78	31.43	35.70
16	15	2	40	6.58	6.48	47.70	50.30
17	15	2	35	12.32	8.63	50.16	41.00
18	10	2	45	14.23	12.34	70.23	69.00
19	10	2	35	13.21	11.16	44.99	42.00
20	0	2	40	14.65	15.70	65.81	68.00
21	15	2	45	14.65	15.90	65.81	67.00
22	5	2	35	14.65	15.66	65.81	70.00

**FIGURE 6:**
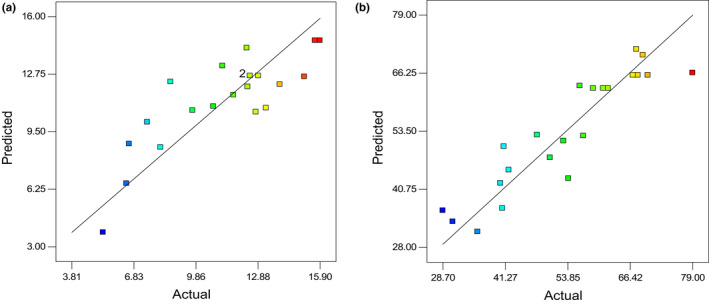
Actual values versus predicted values of BR (a) and HRY (b)

**FIGURE 7:**
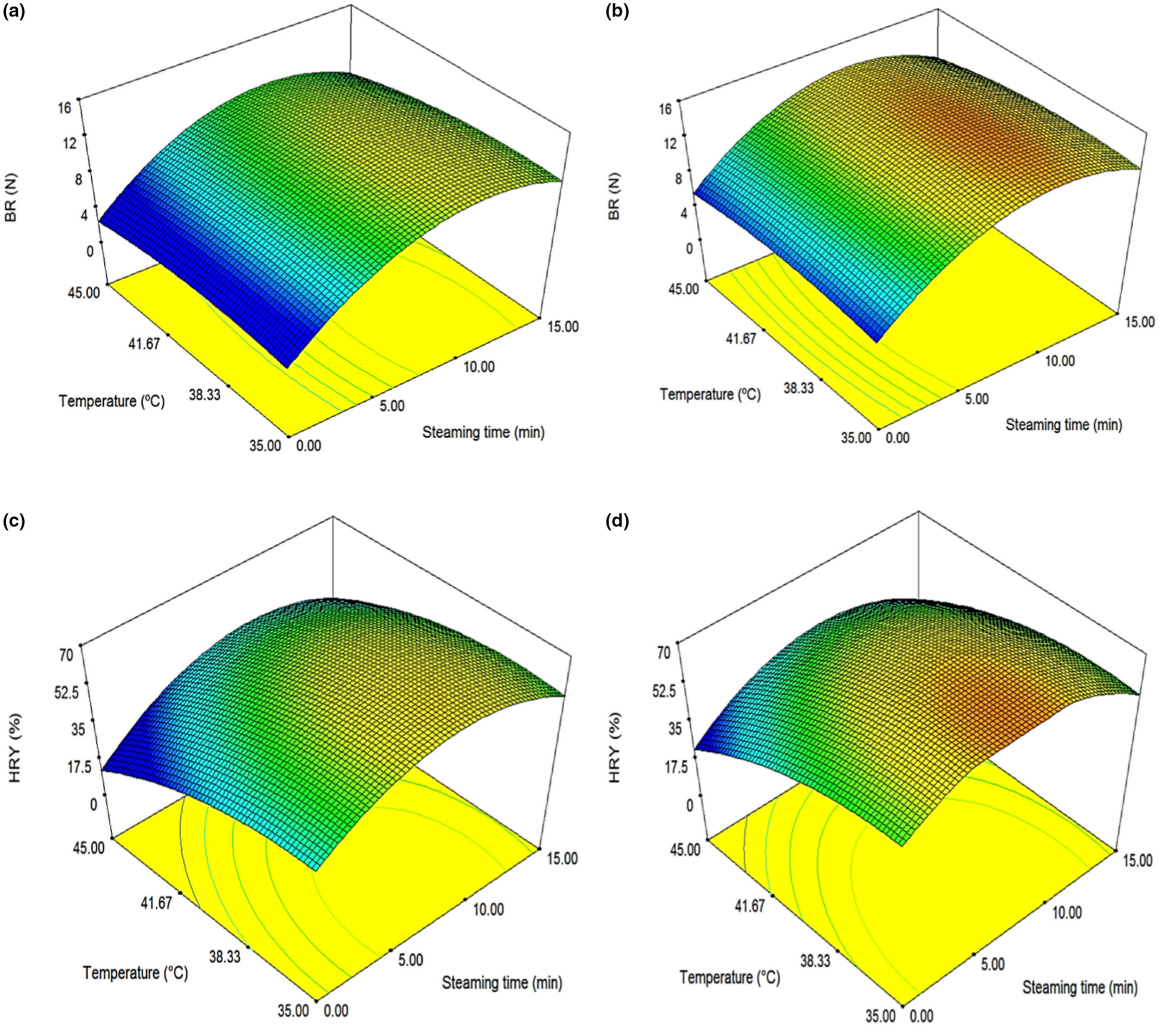
RSM surface plots of BR and HRY: (a,c) solar dryer and (b,d) continuous dryer

Figure 6 indicates a relatively strong correlation between the predicted and experimental values of BR and HRY, with a high value of determination coefficient (*R* ^2^) .984 and .999, respectively. A similar study was performed by Ogunbiyi et al. wherein the RSM method was exclusively used for optimization of HYR and broken rice ratio. Examination of the observed and predicted values of HRY confirmed that the model can sufficiently predict the HRY for the parboiling factors. The adequacy of the model was further confirmed by the determination coefficient (*R* ^2^) and *R* ^2^ (adjusted) values (Ogunbiyi et al., 2018). The three‐dimensional response surface plot of graphical interface for regression equation of reaction variables is shown in Figure 7.

**FIGURE 8:**
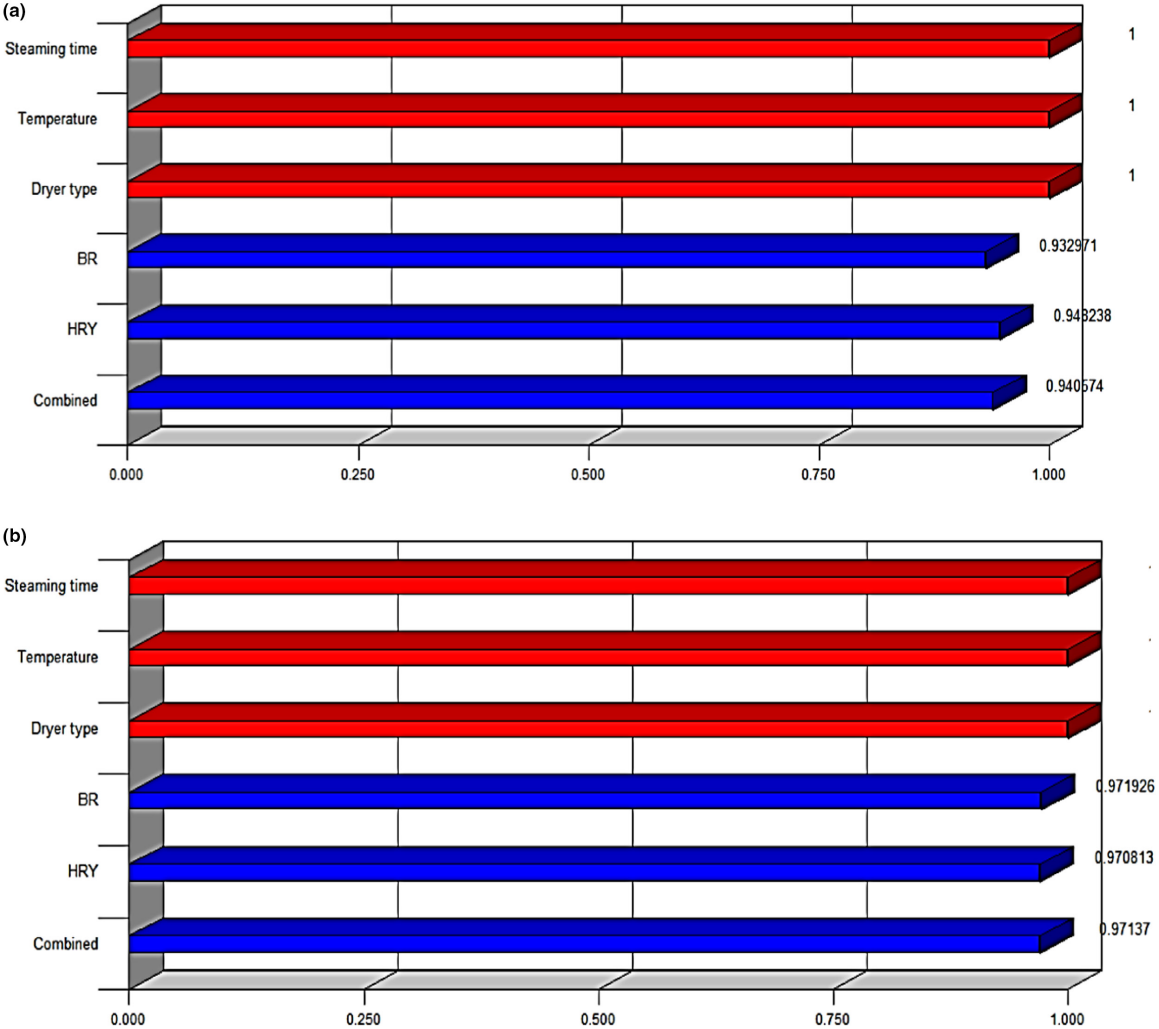
Optimization results based on desirability: (a) solar dryer and (b) continuous dryer

The 3D plots were also designed to evaluate the interactive effects of the independent variables. It is designed by plotting the response variable (HRY and BR) on the z‐axis against independent variables while maintaining other variables at their optimal levels. The 3D plots are helpful for understanding both main and other interactive effects of the three factors. Figure 7 shows the surface plots of rice performance characteristics. The interaction between two independent variables on BR and HRY parameter is illustrated in plots from (a) to (d). Interactions of each independent variable on the response variables are plotted in surface plots using regression equation. Plot (a,b) reveals the significant interaction effect between steaming time (min) and drying air temperature (°C) on the changes in the BR is clearly observable in the plot; that is, the BR at first increases significantly by increasing both steaming time and drying air temperature at both dryer types. Also, there is an exponential increase in BR for increase in steaming time at 10 min at both dryers. According to Figure 7a, the highest value of BR for the solar dryer was obtained as 15.14 N at a drying air temperature of 35°C. On the other hand, Figure 7b shows the highest value of BR for the continuous dryer as 15.9 N at a drying air temperature of 40°C. The maximum increase of BR was found to be 183.5% and 145.4% for the solar and continuous dryer, respectively. Therefore, the continuous dryer showed more BR values compared to the solar dryer because of overturning the rice grain on continuous dryer bed. In addition, their local movement orbital motion of the grains on the seed bed increased their chances to aerate from all directions. Continuous bed provides the possibility of accelerating the drying rate than fix bed in solar dryer (Nassiri & Etesami, 2011). Plot (c,d) presents the nature of steaming time and drying air temperature on the HRY. In addition, it can be observed a proportional increase in the HRY as the steaming time increases, whereas a slight increase can be seen when the drying air temperature is increased at 10 min steaming time and drying air temperature of 35°C and 40°C for solar and continuous dryer, respectively. According to Figure 7c, the highest value of HRY for solar dryer was obtained as 68.7% at a drying air temperature of 35°C. On the other hand, Figure 7d shows the highest value of HRY for continuous dryer as 70% at a drying air temperature of 40°C. The maximum increasing of HRY was found to be 139.4% and 96.1% for solar and continuous dryer, respectively. Thus, the continuous dryer showed more HRY values compared to the solar dryer because of more bending strength of rice grains dried by the continuous dryer. This phenomenon occurs due to alternative heating periods while unloading and reloading the dryer. Dong et al. (2010) reported that tempering has a significant influence on the moisture content gradient in kernels. Tempering reduces heat fluxes and avoids crack development within kernels, resulting in increased HRY (Dong et al., 2010). Eventually, to reach the optimization condition to maximize the BR and HRY values, the predicted conditions are as given in Table 4. The optimum values of BR and HRY were obtained as 12.80 N and 67.3%, respectively, at 9.62 min and 36.9°C for the solar dryer with a desirability of 0.941. In addition, the same values were obtained as 14.50 N and 72.1%, respectively, at 8.77 min and 37.0°C for the continuous dryer with a desirability of 0.971. Separate and combined optimized solutions for each response with their desirability are shown for the solar dryer (Figure 8a) and the continuous dryer (Figure 8b). The best output was related to the HRY and BR for solar and continuous dryers, respectively. A test was performed comparing the optimized BR and HRY values with their actual values to assess the validation for both dryers. The responses showed the low error rates (under 10%). Table 5 indicates the optimization responses. The results showed that RSM could adequately model the rice parboiling process for maximum HRY and BR. The model successfully defined the degree of influence of each studied variable on the response variable. Therefore, RSM can be used to optimize the parboiling process for optimum HRY and BR. Danbaba et al. (2014) used RSM involving central composite design (CCD) to study the effects of soaking temperature, steaming time, and drying time on the HRY of parboiled rice. The coefficient of determination *R* ^2^ and adjusted *R* ^2^ were .97 and .91, respectively, indicating the appropriateness of the model equation in predicting head rice recovery when the three processing variables are mathematically combined (Danbaba et al., 2014). Prediction of HRY and BR under various processing conditions can be improved to commercial production quality and effective policymakers and other decisionmakers in the field of rice production. In this study, the high *R* ^2^ and low RMSE values for the HRY and BR variables showed that the ANN and RSM models can adequately predict and optimize the outputs, but more experimental data need to be gathered to obtain better accuracy of these parameters. In addition, it can be tested the accuracy of other modeling methods such as adaptive network‐based fuzzy inference system and support vector regression to analyze rice parboiling process.

**TABLE 4:** Predicted conditions to reach optimum rice performance characteristics

Number	Steaming time (min)	Dryer type	Temperature (°C)	BR (*N*)	HRY (%)	Desirability
**1**	9.62^*^	1^*^	36.88^*^	12.80^*^	67.3^*^	0.941^*^
2	9.60	1	36.84	12.78	67.1	0.940
3	9.57	1	36.81	12.75	66.8	0.938
4	9.54	1	36.77	12.72	66.5	0.937
5	9.49	1	36.73	12.68	66.3	0.935
**6**	8.77^*^	2^*^	36.97^*^	14.50^*^	72.07^*^	0.971^*^
7	8.74	2	36.94	14.47	72.02	0.970
8	8.71	2	36.92	14.45	71.96	0.969
9	8.69	2	36.87	14.43	71.91	0.966
10	8.65	2	36.85	14.41	71.87	0.965

^*^ Signiﬁcant at 1% probability level.

**TABLE 5:** Verification criteria of optimized responses based on error percentage

ST (min)	Temp (°C)	DT	Value	BR (*N*)	HRY (%)
9.62	36.9	SD	Actual	13.73	68.1
			predicted	12.80	67.3
			Error (%)	6.77	1.17
8.77	37.0	CD	Actual	15.37	72.9
			predicted	14.50	72.1
			Error (%)	5.66	1.10

Abbreviations: CD, Continuous dryer; SD, Solar dryer.

## 4 CONCLUSIONS

This study aimed at developing LR, MLP, and RBF models to predict and determine optimal output variables (BR and HRY) of Hashemi cultivar rice under different parboiling condition (steaming time, dryer type, and inlet drying air temperature). Several conclusions were drawn as follows:

1. Development of LR model for BR (*R* ^2^ = .282) and HRY (*R* ^2^ = .664) illustrated unsuitable correlation for the relationships between the measured and predicted values. The impacts of steaming time, dryer type, and inlet drying air temperature are statistically significant for HRY and BR (*p* < .05).
2. The coefficient of determination was applied to estimate the predictive performance of the ANN models for the measured and predicted values. The *R* ^2^ values were determined as .983 and .981 for BR and HRY, respectively. This result showed that the predictive accuracy of the ANN model for both output variables were high. With respect to observed cross‐correlation among predicted and target values, it could be detected that MLP model was effective for predicting HRY and BR of parboiled rice.
3. It is concluded that RBF can provide a useful predictive tool for the estimation of rice HRY and BR parameters. Based on achieved parameters of the final chosen model, the RBF model shows higher acceptability for the prediction of rice HRY (*R* ^2^ = .986; RMSE = .041) and BR (*R* ^2^ = .989; RMSE = .043) than that of the MLP ones. It can be observed that RBF network can be used with almost the same accuracy and higher efficiency to model and predict output data than the MLP models.
4. The optimum values of BR and HRY were obtained as 12.80 N and 67.3%, respectively, at 9.62 min and 36.9°C for solar dryer with desirability of 0.941. In addition, the same values were obtained as 14.50 N and 72.1%, respectively, at 8.77 min and 37.0°C for the continuous dryer with a desirability of 0.971. From the results, it can be concluded that RSM can adequately model rice parboiling processing for maximum HRY and BR.
5. In general, results indicated that RSM, LR, and ANN could be successfully used to describe experimental data. All methods exhibited certain advantages, that is, ANN showed minor advantage of fitting quality, while RSM provided further insights into optimization of parboiling conditions.

## ACKNOWLEDGEMENTS

The authors would like to thank Shiraz University for its facilities and technical support.
